# Residual dipolar line width in magic-angle spinning proton solid-state NMR

**DOI:** 10.5194/mr-2-499-2021

**Published:** 2021-07-01

**Authors:** Matías Chávez, Thomas Wiegand, Alexander A. Malär, Beat H. Meier, Matthias Ernst

**Affiliations:** Physical Chemistry, ETH Zürich, Vladimir-Prelog-Weg 2, 8093 Zürich, Switzerland

## Abstract

Magic-angle spinning is routinely used to average anisotropic interactions in solid-state nuclear magnetic resonance (NMR). Due to the fact that the homonuclear dipolar Hamiltonian of a strongly coupled spin system does not commute with itself at different time points during the rotation, second-order and higher-order terms lead to a residual dipolar line broadening in the observed resonances. Additional truncation of the residual broadening due to isotropic chemical-shift differences can be observed. We analyze the residual line broadening in coupled proton spin systems based on theoretical calculations of effective Hamiltonians up to third order using Floquet theory and compare these results to numerically obtained effective Hamiltonians in small spin systems. We show that at spinning frequencies beyond 75 kHz, second-order terms dominate the residual line width, leading to a $1/\omega_{r}$ dependence of the second moment which we use to characterize the line width. However, chemical-shift truncation leads to a partial $\omega_{r}^{-2}$ dependence of the line width which looks as if third-order effective Hamiltonian terms are contributing significantly. At slower spinning frequencies, cross terms between the chemical shift and the dipolar coupling can contribute in third-order effective Hamiltonians. We show that second-order contributions not only broaden the line, but also lead to a shift of the center of gravity of the line. Experimental data reveal such spinning-frequency-dependent line shifts in proton spectra in model substances that can be explained by line shifts induced by the second-order dipolar Hamiltonian.

## 1 Introduction

Magic-angle spinning (MAS) [Bibr bib1.bibx4] is a prerequisite for almost all high-resolution solid-state nuclear magnetic resonance (NMR) spectroscopic techniques. Over the years, a steady increase in MAS frequencies has been achieved [Bibr bib1.bibx7] up to 170 kHz for cylindrical rotors [Bibr bib1.bibx24], and alternate rotor designs have also been implemented based on spheres [Bibr bib1.bibx13]. With the spinning frequencies currently available, proton-detection experiments in fully protonated and labeled proteins have become feasible [Bibr bib1.bibx2]. The residual line width of such proton-detected protein spectra is on the order of a few hundred Hertz (full width at half maximum, FWHM) and decreases with increasing spinning frequency, $\omega_{r}$ . There are different contributions to the residual line width as discussed in detail in [Bibr bib1.bibx36], which can be classified as homogeneous or inhomogeneous contributions according to [Bibr bib1.bibx29]. The inhomogeneous contributions can be refocused in a Hahn echo experiment [Bibr bib1.bibx20], e.g., magnetic-field inhomogeneity or susceptibility broadening, while homogeneous contributions cannot be refocused. The homogeneous contributions originate either from coherent terms that are due to incomplete averaging by MAS or from incoherent relaxation due to stochastic dynamic processes in the molecule. The incoherent relaxation contribution is expected to change only little with a change in MAS frequency since the only difference comes from the spinning-frequency-dependent sampling of the spectral-density function $J(\omega_{r})$ [Bibr bib1.bibx39] that contributes to $T_{2}$ . The refocused line width $\mathrm{FWHM}(\mathrm{hom})=\pi /T_{2}^{\prime }$ is, therefore, the sum of a nearly constant term $\pi /T_{2}$ and the coherent contribution that scales with the spinning frequency.

To describe experiments with time-dependent Hamiltonians as is the case under MAS, average-Hamiltonian theory (AHT) [Bibr bib1.bibx19] or Floquet theory [Bibr bib1.bibx41] can be used to calculate effective time-independent Hamiltonians to different orders. Since the dipolar coupling is a traceless second-rank tensor, one would expect no contribution in the first-order term. However, second-order (commutator) terms and third-order (double-commutator) terms are possible as well as higher-order contributions. The second-order terms are expected to scale with $1/\omega_{r}$ , while in general terms on order n are expected to scale with $\omega_{r}^{-(n-1)}$ . Experimental observations of the residual homogeneous line width as a function of spinning frequency show that it can often be approximated by a linear correlation with the inverse of the spinning frequency with some deviation that indicates a partial inverse quadratic dependence [Bibr bib1.bibx34]. This has been attributed to third-order contributions to the effective Hamiltonian or to chemical-shift effects [Bibr bib1.bibx45].

Second-order effective Hamiltonians under MAS for strongly coupled spin systems have been calculated before based on AHT [Bibr bib1.bibx11] for three-spin sub-systems with an arbitrary geometry. We extend this work to a general solution for the third-order terms based on Floquet theory. The analytical solutions can be used to calculate spectra numerically based on different orders of the analytical solution and analyze their scaling behavior under MAS. Alternatively, exact effective Hamiltonians based on the numerical propagator (calculated by time slicing of the time-dependent Hamiltonian) can be calculated using the matrix logarithm of the propagator [Bibr bib1.bibx25]. When calculating an effective Hamiltonian from the propagator using the matrix logarithm, one has to keep in mind that the eigenvalues can only be determined modulo the inverse of the cycle time, which is in our case the spinning frequency. For fast spinning, i.e., short cycle times, this is usually not a problem since the eigenvalues of the effective Hamiltonian are within the interval $\pm \omega_{r}/2$ . Based on these different effective Hamiltonians, we calculate the second moment ( $M_{2}$ ) of the line which can be correlated with an equivalent line width of a Gaussian line [Bibr bib1.bibx32].

We show that third-order terms do not play a critical role in the residual line width at MAS spinning frequencies beyond 75 kHz. At slower spinning frequencies, cross terms between chemical shifts and homonuclear dipolar couplings start to play an important role in third-order terms. Due to the structure of the second-order Hamiltonian, the lines are not only broadened, but also shifted, which can be characterized by the first moment ( $M_{1}$ ) of the line which describes the center of gravity of the line. These line shifts can be observed experimentally. The experimentally observed deviation from the expected $1/\omega_{r}$ dependence can be reproduced in numerical calculations only if chemical-shift differences are considered. The additional truncation of the residual dipolar couplings by the chemical shift is the reason for this difference.

## 2 Theory

We assume a homonuclear spin system with chemical shifts and homonuclear dipolar couplings under MAS. The time-dependent Hamiltonian for such a system can be written as

1 $$\begin{array}{rl} \hat{H}(t) & =\sum_{p=1}^{N}\omega_{p}{\hat{I}}_{pz}+\sum_{p=1}^{N-1}\sum_{q=p+1}^{N}\omega_{pq}(t)\\ & \times \left[2{\hat{I}}_{pz}{\hat{I}}_{qz}-\left({\hat{I}}_{px}{\hat{I}}_{qx}+{\hat{I}}_{py}{\hat{I}}_{qy}\right)\right], \end{array}$$

where the time-dependent dipolar coupling is given by

2 $$\omega_{pq}(t)=\sum_{m=-2}^{2}\omega_{pq}^{\left(m\right)}e^{im\omega_{r}t},$$

and the Fourier coefficients are defined by

3 $$\begin{array}{rl} \omega_{pq}^{\left(m\right)} & =\frac{1}{\sqrt{6}}\sum_{m=2}^{2}\sum_{m^{\prime }=-2}^{2}d_{m,0}^{2}\left(-\theta_{m}\right)e^{-im\gamma }d_{m^{\prime },m}^{2}(\beta )e^{-im^{\prime }\alpha }\\ & \times e^{-im^{\prime }\phi_{pq,12}}d_{0,m^{\prime }}^{2}\left(\theta_{pq,12}\right)\sqrt{\frac{3}{2}}\delta_{pq}. \end{array}$$

Here, $\delta_{pq}$ is the anisotropy of the dipolar coupling and $d_{m,m^{\prime }}^{l}(\theta )$ are the reduced Wigner rotation matrix elements. We transform the dipolar-coupling tensor from the principal-axes system (PAS) to the laboratory frame system (LAB) by three consecutive Euler rotations: (i) a rotation by $\left(0,\theta_{\left(pq\right)},\phi_{\left(pq\right)}\right)$ from the PAS of spin pair (pq) to the PAS of spin pair (12) ; (ii) a rotation by (α,β,γ) from the PAS of spin pair (12) to the rotor fixed frame; (iii) a rotation by $\left(-\omega_{r}t,-\theta_{m},0\right)$ from the rotor-fixed frame to the laboratory frame of reference. The Hamiltonian is periodic with a single frequency and can be written as a Fourier series

4 $$\hat{H}(t)=\sum_{n=-2}^{2}{\hat{H}}^{\left(n\right)}e^{in\omega_{r}t},$$

and the Fourier coefficients of the Hamiltonian are given by

5H^(0)=∑p=1NωpI^pz,6H^(n)=∑p=1N-1∑q=p+1Nωpq(n)2I^pzI^qz-I^pxI^qx+I^pyI^qy.

Based on single-mode Floquet theory, we can now calculate the first three orders of the effective Hamiltonian [Bibr bib1.bibx41] according to

7 $$\begin{array}{rl} \hat{\bar{H}} & ={\hat{\bar{H}}}^{\left(1\right)}+{\hat{\bar{H}}}^{\left(2\right)}+{\hat{\bar{H}}}^{\left(3\right)}+\cdots \\ & ={\hat{H}}^{\left(0\right)}+\frac{1}{2}\sum_{n\neq 0}\frac{\left[{\hat{H}}^{\left(n\right)},{\hat{H}}^{\left(-n\right)}\right]}{n\omega_{r}}\\ & +\frac{1}{2}\sum_{n\neq 0}\frac{\left[\left[{\hat{H}}^{\left(n\right)},{\hat{H}}^{\left(0\right)}\right],{\hat{H}}^{\left(-n\right)}\right]}{{\left(n\omega_{r}\right)}^{2}}\\ & +\frac{1}{3}\sum_{k,n\neq 0}\frac{\left[{\hat{H}}^{\left(n\right)},\left[{\hat{H}}^{\left(k\right)},{\hat{H}}^{\left(-k-n\right)}\right]\right]}{n\omega_{r}k\omega_{r}}+\cdots \end{array}$$

Second-order terms are fully described by a three-spin system, while third-order terms require a four-spin system to obtain all possible terms. The first three orders of the effective Hamiltonian for a dipolar-coupled spin system are given by

8H‾^(1)=H^(0)=∑p=1NωpI^pz,9H‾^(2)=12∑n≠0H^(n),H^(-n)nωr=∑p,q,rωpqreffI^pzI^q+I^r--I^q-I^r+10H‾^(3)=12∑n≠0H^(n),H^(0),H^(-n)nωr2+13∑k,n≠0H^(n),H^(k),H^(-k-n)nωrkωr,=∑pωpeffI^pz+∑p,qωpq,zeff2I^pzI^qz-ωpqeffI^pxI^qx+I^pyI^qy+∑p,q,rωpqreffI^pzI^q+I^r-+I^q-I^r++∑p,q,r,sωpqrs,zeffI^pzI^qzI^r+I^s-+I^r-I^s++∑p,q,r,sωpqrs,xy(eff)I^p+I^q-I^r+I^s-+I^p-I^q+I^r-I^s+.

Detailed expressions for the various effective coupling frequencies in Eqs. (9) and (10) can be found in the Supplement or for the second-order terms in [Bibr bib1.bibx11].

The second-order Hamiltonian is a three-spin zero-quantum-type Hamiltonian with an additional ${\hat{I}}_{z}$ term as the third spin operator. Time evolution under such a zero-quantum Hamiltonian does not lead to line splitting, but a line shift has been shown for the rank-one part of the J coupling [Bibr bib1.bibx3], which also has a purely zero-quantum Hamiltonian [Bibr bib1.bibx32]. This fact has also been mentioned in a recent paper about homonuclear J decoupling in solids [Bibr bib1.bibx33]. As a consequence of this, the second-order Hamiltonian will not only lead to a line broadening, but also a shift of the line that depends on the spinning frequency. In the presence of large chemical-shift differences, the second-order Hamiltonian will be completely truncated and becomes invisible in the limit of weak coupling. The third-order Hamiltonian, however, also contains terms that lead to a splitting of the lines and are visible in the weak-coupling limit.

## 3 Numerical simulations

All numerical simulations have been implemented using the spin-simulation environment GAMMA [Bibr bib1.bibx43], which allows the use of arbitrary effective Hamiltonians. To characterize the line width in a homonuclear dipolar-coupled spin system, two different approaches were used. In a first approach, the propagator of the time-dependent Hamiltonian over a full rotor cycle was calculated using time slicing of the Hamiltonian. The effective Hamiltonian over the rotor cycle was then back-calculated according to

11 $$\hat{\bar{H}}=\frac{\mathrm{ln}\left(U(\tau_{r})\right)}{i2\pi \tau_{r}}.$$

Here, $\tau_{r}=2\pi /\omega_{r}$ is the cycle time of the MAS rotation. Typically, one has to be careful that the eigenvalues of the Hamiltonian can be multivalued with a multiple of $\omega_{r}=\frac{2\pi }{\tau_{r}}$ and the correct solution is unknown. In the case of fast MAS, however, the eigenvalues of the effective Hamiltonian are typically much smaller than the spinning frequency, and this multivalued solution of the logarithm poses no problems. The second approach uses the series expansion of the effective Hamiltonian based on Floquet theory as presented in the Theory section. This allows us to compare spectra or properties of spectra as a function of the different levels of approximation as provided by Floquet theory. We diagonalize the effective Hamiltonian obtained from either method and calculate transition frequencies ( $\omega_{ij}$ ) from the difference of the eigenvalues of the Hamiltonian in the eigenbase. The initial density operator (usually ${\hat{F}}_{x}=\sum_{n}{\hat{I}}_{nx}$ ) and the detection operator (usually ${\hat{F}}^{-}=\sum I_{nx}-i\sum_{n}I_{ny}$ ) are transformed into the eigenbase of the Hamiltonian, and the off-diagonal elements are used to determine the intensity of the transitions ( $I_{ij}=(\sigma_{0}{)}_{ij}(d_{ji}{)}^{*}$ ). Here, $\sigma_{0}$ is the initial density operator and d is the detection operator. This is basically a standard frequency-based spectrum calculation that allows us to reconstruct the spectrum with any desired resolution. Either spectra are calculated by binning the transition frequencies into a spectral range with a given frequency resolution or the intensities, and the frequencies are directly used to calculate the n th moment of the line according to

12 $$M_{n}=\frac{\sum_{i,j<i}\omega_{ij}^{n}I_{ij}}{\sum_{i,j<i}I_{ij}}.$$

The second method has a higher precision because the rounding due to the binning of the frequency values is avoided. Instead of the second moment ( $M_{2}$ ), we use the equivalent FWHM of a Gaussian line with the same $M_{2}$ , which is given by $\mathrm{FWMH}=2\sqrt{2\mathrm{ln}(2)M_{2}}$ as a measure of the line width [Bibr bib1.bibx32].

## 4 Results and discussions

The spin systems are characterized by the coordinates of the spins $r_{i}=(x_{i},y_{i},z_{i})$ leading to the distances $r_{ij}$ and a set of Euler angles $\left(0,\theta_{ij},\phi_{ij}\right)$ . The anisotropy of the dipolar coupling is then given by $\delta_{ij}=-2\frac{\mu_{0}}{4\pi }\frac{\gamma_{i}\gamma_{j}\hbar }{r_{ij}^{3}}$ . We start with discussing a three-spin system since many characteristics of the residual line width can already be seen in this simple spin system. We assume the spin pair (12) is aligned along the z axis defined by the static magnetic field. In a planar three-spin system, a single Euler angle is sufficient to describe the orientation of each coupling with respect to the coupling (12). Figure [Fig Ch1.F1]a shows the geometry of the three-spin system used in the simulations. In a second step we will go to a four-spin system where the fourth spin can either be in or out of the plane spanned by the spins 1, 2, and 3 (see Fig. [Fig Ch1.F1]b). The details of each spin system are always given in the figure captions.

**Figure 1 Ch1.F1:**
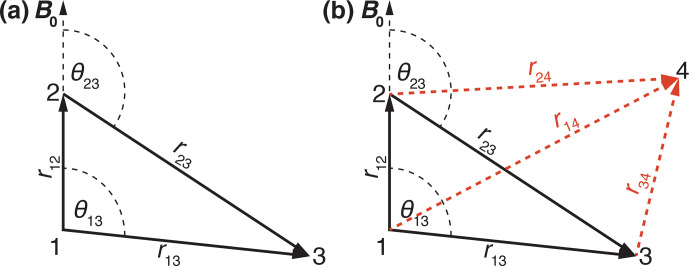
Schematic drawing of the **(a)** three-spin and **(b)** four-spin systems used in numerical simulations indicating distances and relative orientations. The red dashed lines indicate out-of-plane vectors to the fourth spin.

### 4.1 Three-spin system without chemical-shift differences

For simplicity, we start out with a three-spin system without chemical-shift differences and coordinates $r_{1}=(0,0,0)$ , $r_{2}=(0,0,1.75)$ , and $r_{3}=(3,0,0)$ (in units of Å) leading to distances and angles given by $r_{12}$ = 1.75 Å, $r_{13}$ = 3.00 Å, $r_{23}$ = 3.47 Å, $\theta_{13}$ = 90 ${}^{\circ }$ , and $\theta_{23}$ = 120 ${}^{\circ }$ (see Fig. [Fig Ch1.F1]). This is a mimic for a strongly coupled ${\mathrm{CH}}_{2}$ spin system with one additional more distant proton. The dipolar couplings in such a spin system are characterized by $\delta_{12}/(2\pi )=-44\;826$ Hz, $\delta_{13}/(2\pi )=-8898$ Hz, $\delta_{23}/(2\pi )=-5750$ Hz. Figure [Fig Ch1.F2]a shows the calculated spectra for this spin system at an MAS frequency of 50 kHz using second-order, third-order, and exact effective Hamiltonians. Note that the spectrum is cut off in the center at the top, and the complete spectrum is shown as an inset in Fig. [Fig Ch1.F2]a. Spectra at other spinning frequencies ranging from 20 kHz to the currently experimentally inaccessible 1000 kHz can be found in the Supplement (Fig. S1). An expanded view of the central part of the spectra is shown in Fig. [Fig Ch1.F2]b. The second-order Hamiltonian leads to a spectrum (red) that is already quite close to the correct spectrum obtained from the numerically calculated effective Hamiltonian (blue) with some differences in features in the center part of the spectrum (see Fig. [Fig Ch1.F2]b). These differences are strongly reduced when we include the third-order terms (orange), which leads to a spectrum that has all the features and is very close to the exact one (blue) in the center of the line. The second-order and third-order spectra, however, agree very well outside the central region. The fact that the third-order Hamiltonian leads only to changes in the central part of the spectrum can be understood from the form of the Hamiltonian as shown in Eqs. (9) and (10). Since the two Hamiltonians do not commute, a large value for ${\hat{\bar{H}}}^{\left(2\right)}$ will truncate ${\hat{\bar{H}}}^{\left(3\right)}$ , leading to negligible changes in the spectrum at larger offsets. In the center of the spectrum ${\hat{\bar{H}}}^{\left(2\right)}$ is smaller, and the influence of ${\hat{\bar{H}}}^{\left(3\right)}$ is visible in the spectra.

**Figure 2 Ch1.F2:**
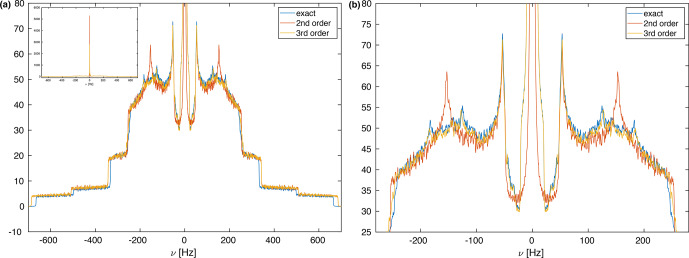
**(a)** Calculated MAS spectra at a spinning frequency of 50 kHz using different approximations of the effective Hamiltonian. All spectra were processed with an exponential line broadening of 1 Hz. The central very narrow peak is cut for a better display of the spectrum, but the complete spectrum is shown as an inset in the upper left corner. The spin-system parameters are $\delta_{12}/(2\pi )=-44\;826$ Hz, $\delta_{13}/(2\pi )=-8898$ Hz, $\delta_{23}/(2\pi )=-5750$ Hz, $\theta_{13}=90$ ${}^{\circ }$ , $\theta_{23}=120$ ${}^{\circ }$ . The spectral window was set to 2000 Hz with 20 000 data points leading to a digital resolution of 0.1 Hz. One hundred thousand powder points were sampled according to the ZCW scheme [Bibr bib1.bibx14]. **(b)** Expanded central region of the spectrum in panel **(a)**. Here one can clearly see the differences between the second-order and third-order approximations that are very close to the exact spectrum.

However, there are clear differences in the line width of the exact spectrum and the spectrum based on second-order and third-order effective Hamiltonians, which are clearly broader. This difference in the breadth of the powder line shape must be attributed to higher-order contributions to the effective Hamiltonian. Since calculating terms beyond the third-order term considered here are quite complex, we have investigated how the difference of the total breadth scales with spinning frequency. Figure [Fig Ch1.F3] shows the difference in breadth in a double-logarithmic plot for spinning frequencies ranging from 20 to 250 kHz. The data points lie on a line with slope -3 , which indicates that the breadth depends on the spinning frequency with the inverse third power. This is a clear indication that the additional term that leads to the difference in breadth is a fourth-order effective Hamiltonian term that would scale with $\omega_{r}^{-3}$ .

**Figure 3 Ch1.F3:**
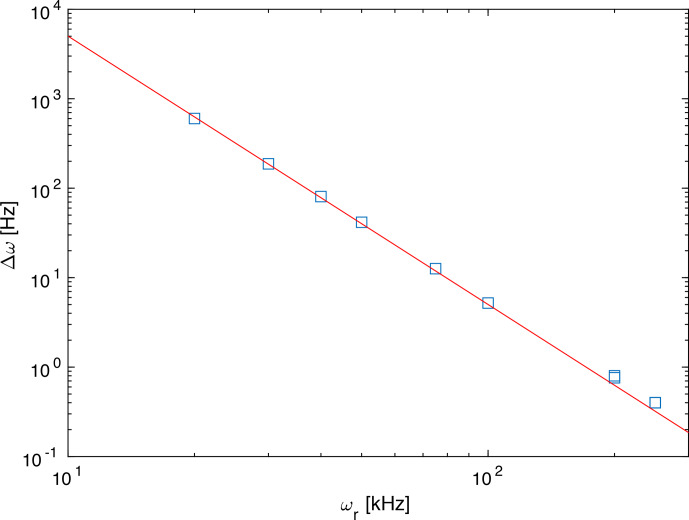
Difference in breadth of the simulated spectra between the third-order and exact effective Hamiltonians as a function of the spinning frequency. The red line has a slope of -3 corresponding to a $\omega_{r}^{-3}$ dependence of the difference. This is the expected slope for a contribution by a fourth-order effective Hamiltonian.

We can calculate the second moment of the powder lines as a function of the spinning frequency (see Fig. S1) as shown in Fig. [Fig Ch1.F4]. Above a spinning frequency of 50 kHz, there is a very good linear correlation between the line width and the inverse spinning frequency ( $\omega_{r}^{-1}$ ) for all three sets of spectra. For second-order and third-order spectra, the correlation extends down to 20 kHz MAS frequency, but for the full effective Hamiltonian, we observe a deviation from the linear correlation. This deviation towards narrower lines is most likely due to the fourth-order effective Hamiltonian as discussed above. This deviation becomes important for spinning frequencies below 50 kHz, where the spinning frequency is on the order of the biggest dipolar coupling used in the model spin system. It is well known that perturbation expansions such as the average Hamiltonian or the van Vleck expansion converge slowly in this regime [Bibr bib1.bibx6]. It is interesting to note that the line widths for the second-order (red) and third-order (orange) spectra agree very well over the full range of spinning frequencies.

**Figure 4 Ch1.F4:**
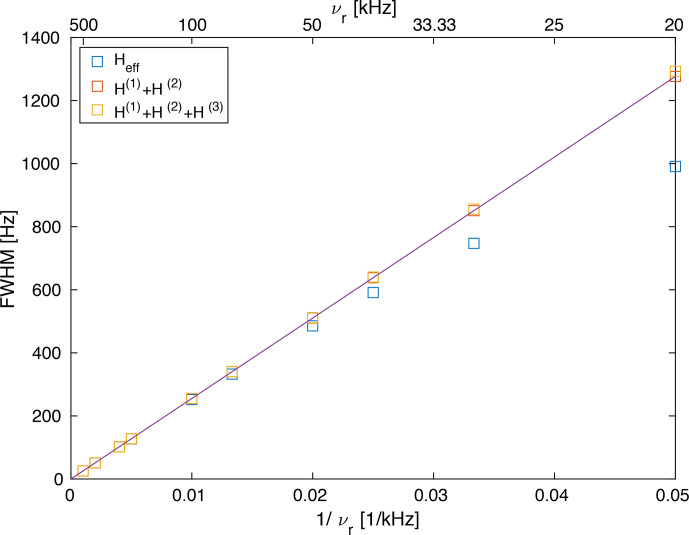
Line width (full width half maximum) of a Gaussian line with the same second moment as the powder line shape shown in Fig. S1 ( $\mathrm{FWMH}=2\sqrt{2\mathrm{ln}(2)M_{2}}$ ). The line width obtained from second-order and third-order effective Hamiltonians correlates almost perfectly linearly with $\omega_{r}^{-1}$ , while the line width obtained from the exact effective Hamiltonian shows some deviations which become more prominent below 50 kHz. This deviation (narrower line width) is the contribution of the fourth-order term as discussed above (see Fig. [Fig Ch1.F3]).

Of course, the second moment calculated over the complete spectrum including the side bands is preserved under MAS and is independent of the spinning frequency [Bibr bib1.bibx26]. Since we are interested in the line width of the center band, the second moment is calculated only over the center band of the line, and we see a decrease in the second moment as a function of the spinning frequency and a corresponding decrease in the Gaussian line width.

### 4.2 Three-spin system with chemical-shift differences

Introducing chemical shifts makes the analysis of the spectra in terms of second moments and line widths more complex. This is due to the fact that we are now interested in second moments of the different lines that may overlap with each other or overlap with combination lines that are possible in strongly coupled spin systems. Figure [Fig Ch1.F5] shows the simulated proton spectra at 100 kHz MAS frequency for two different sets of chemical shifts. Spectra at different spinning frequencies (between 50 and 500 kHz) can be found in Fig. S2. Slower spinning frequencies are difficult to analyze due to overlapping lines. Below the spectra the regions of the various transition frequencies are marked by a black line. One can see that there are single-quantum transitions and combination lines, with the combination lines having much lower intensity than the single-quantum transitions. Calculating the second moment over the complete line and correcting for the contributions by the isotropic chemical shifts confirm that the second moment is preserved, as one would expect. However, this is not the quantity of interest since we are interested in the individual line width. We, therefore, calculate the first and second moments of the individual one-quantum lines by restricting the calculations to the relevant areas of the spectrum indicated in the figure by black lines.

**Figure 5 Ch1.F5:**
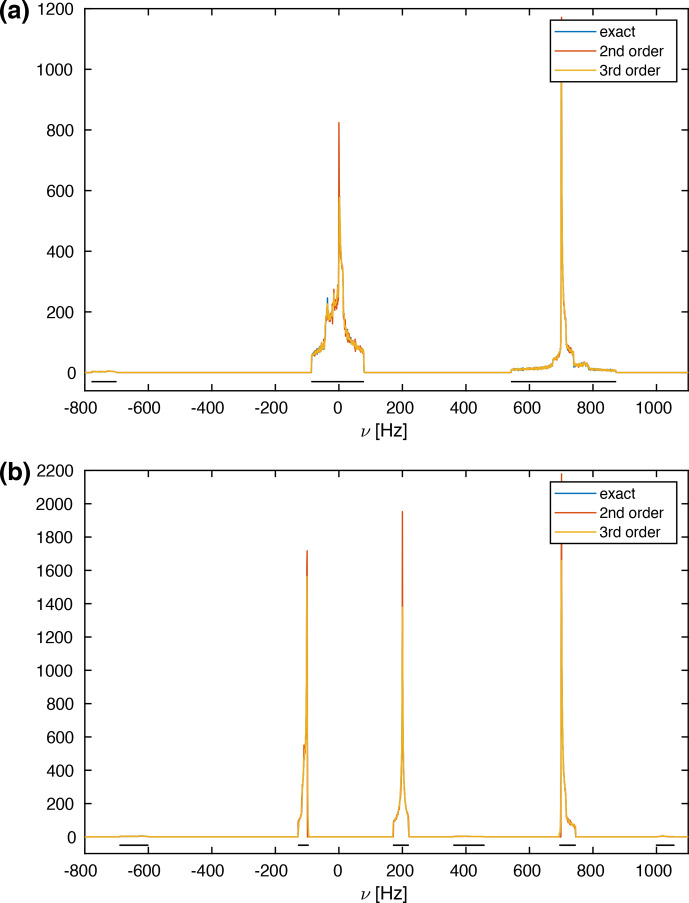
Calculated MAS spectra of a three-spin system at an MAS frequency of 100 kHz. The spin-system parameters are $\delta_{12}/(2\pi )=-44\;826$ Hz, $\delta_{13}/(2\pi )=-8898$ Hz, $\delta_{23}/(2\pi )=-5750$ Hz, $\theta_{13}=90$ ${}^{\circ }$ , $\theta_{23}=120$ ${}^{\circ }$ . The spectral window was set to 20 kHz with 20 000 data points leading to a digital resolution of 1 Hz. One hundred thousand powder points were sampled according to the ZCW scheme [Bibr bib1.bibx14]. The chemical shifts were chosen to be **(a)** $\delta_{1}=\delta_{2}$ = 0 ppm, $\delta_{3}$ = 0.7 ppm and **(b)** $\delta_{1}$ = -0.1 ppm, $\delta_{2}$ = 0.2 ppm, $\delta_{3}$ = 0.7 ppm at a Larmor frequency of 1 GHz. The black line under the spectra indicates regions of transitions.

**Figure 6 Ch1.F6:**
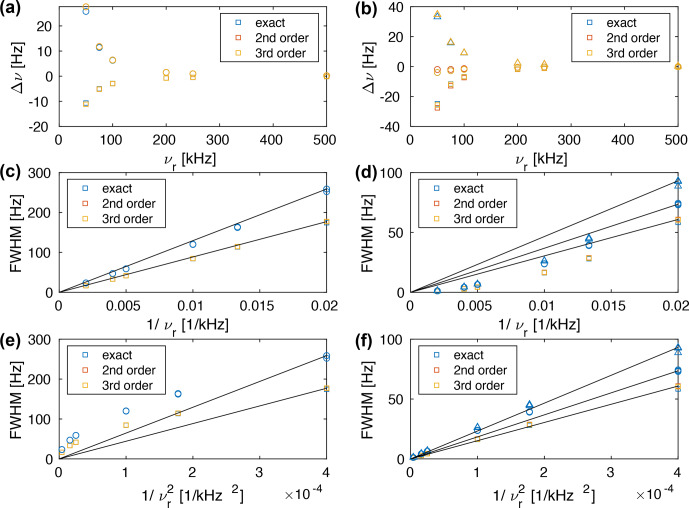
**(a, b)** Plot of the deviation of the calculated chemical shift (first moment of the line) from the theoretical chemical shift. Plot of the line width (FWHM) as a function of **(c, d)** $\nu_{r}^{-1}$ and **(e, f)** $\nu_{r}^{-2}$ . The spin-system parameters are $\delta_{12}/(2\pi )=-44\;826$ Hz, $\delta_{13}/(2\pi )=-8898$ Hz, $\delta_{23}/(2\pi )=-5750$ Hz, $\theta_{13}=90$ ${}^{\circ }$ , $\theta_{23}=120$ ${}^{\circ }$ . The chemical shifts were chosen to be **(a, c, e)** $\delta_{1}=\delta_{2}$ = 0 ppm (squares), $\delta_{3}$ = 0.7 ppm (circles) and **(b, d, f)** $\delta_{1}$ = -0.1 ppm (squares), $\delta_{2}$ = 0.2 ppm (circles), $\delta_{3}$ = 0.7 ppm (triangles) at a Larmor frequency of 1 GHz. The straight lines go through (0,0) and the line width at the slowest spinning frequency. They are meant as a guide to the eye for the linear correlation.

Calculating the transition-selective moments as a function of the MAS frequency shows that the first moment and the second moment are spinning-frequency dependent. The deviation of the first moment from the chemical shift is shown in Fig. [Fig Ch1.F6]a and b. One can see that the differences between the different approximations (second order, third order, and exact effective Hamiltonian) are quite small and that one can observe changes in the line position as a function of the spinning frequency. These changes in the line position will limit the accuracy of the chemical shift determination in proton spectra and will be discussed in more detail in Sect. 5. The dependence of the line width on the spinning frequency is shown in Fig [Fig Ch1.F6]c–f for chemical shifts $\delta_{1}=\delta_{2}$ = 0 ppm (squares), $\delta_{3}$ = 0.7 ppm (circles) (left column) and $\delta_{1}$ = - 0.1 ppm (squares), $\delta_{2}$ = 0.2 ppm (circles), $\delta_{3}$ = 0.7 ppm (triangles) (right column) as a function of $\nu_{r}^{-1}$ (middle row), and $\nu_{r}^{-2}$ (lower row). One can see that for three-spin systems with chemical-shift differences the clear $\nu_{r}^{-1}$ dependence of the three-spin systems without chemical-shift differences does not hold anymore. For a strongly coupled spin pair with a small chemical shift difference and an additional coupling to a third spin with a strongly different chemical shift (Fig. [Fig Ch1.F6]c and d), the dependence of the line width on the spinning frequency is still quite close to $\nu_{r}^{-1}$ . For a three-spin system with three distinct chemical shifts, the MAS dependence is closer to $\nu_{r}^{-2}$ . In general, the dependence on the MAS frequency is somewhere between $\nu_{r}^{-1}$ and $\nu_{r}^{-2}$ and depends on the exact selection of the chemical shifts. We believe that this change in spinning-frequency dependence is a consequence of additional truncation of the second-order effective Hamiltonian by the chemical shifts. The truncation explains the experimentally observed deviation from the $\nu_{r}^{-1}$ dependence of the homogeneous line width [Bibr bib1.bibx34].

### 4.3 Larger spin system without chemical-shift differences

Figure [Fig Ch1.F1]b shows the geometry of the four-spin system where spin 4 can be out of the plane spanned by spins 1, 2, and 3. Therefore, the directions of $r_{14}$ , $r_{24}$ , and $r_{34}$ are defined by sets of two Euler angles each $\left(0,\theta_{ij},\phi_{ij}\right)$ . For simplicity, we will show simulations for a four-spin system where all four spins are in a plane, i.e., $\phi_{ij}=0$ . Simulations on spin systems with out-of-plane spins show exactly the same behavior. The details of the spin system are given in the figure captions.

Figure [Fig Ch1.F7]a shows spectra for a four-spin system using second-order, third-order, and exact effective Hamiltonians. All three spectra are virtually identical, except for the central sharp peak that is significantly higher in the second-order approximations. In addition, the narrower powder line shapes for the exact effective Hamiltonian are observed as in the three-spin simulations. The second moment of the powder line shape in the four-spin systems scales as a function of the MAS frequency in the same way as for the three-spin systems (see Figs. S3 and S4) almost perfectly with $\omega_{r}^{-1}$ , with some small deviations visible at spinning frequencies below 75 kHz. This behavior is exactly the same as in the three-spin case. If we calculate the second moment of the four-spin system and compare it to the sum of the four three-spin sub-systems, we find $M_{2}^{\left(1234\right)}=\frac{3}{4}\left(M_{2}^{\left(123\right)}+M_{2}^{\left(124\right)}+M_{2}^{\left(134\right)}+M_{2}^{\left(234\right)}\right)$ to within an error of less than 1 % for all spinning frequencies. This allows us to calculate the line width of a multi-spin system based on the three-spin sub-systems as long as the second-order contribution is dominating the residual line width under MAS. The same behavior is observed for five-spin systems that can be decomposed into four-spin or three-spin sub-systems for the calculation of the second moment as long as the second-order contribution dominates the line width (see Figs. S5 and S6). Again, the deviation of the calculated second moments is less than 1 %.

**Figure 7 Ch1.F7:**
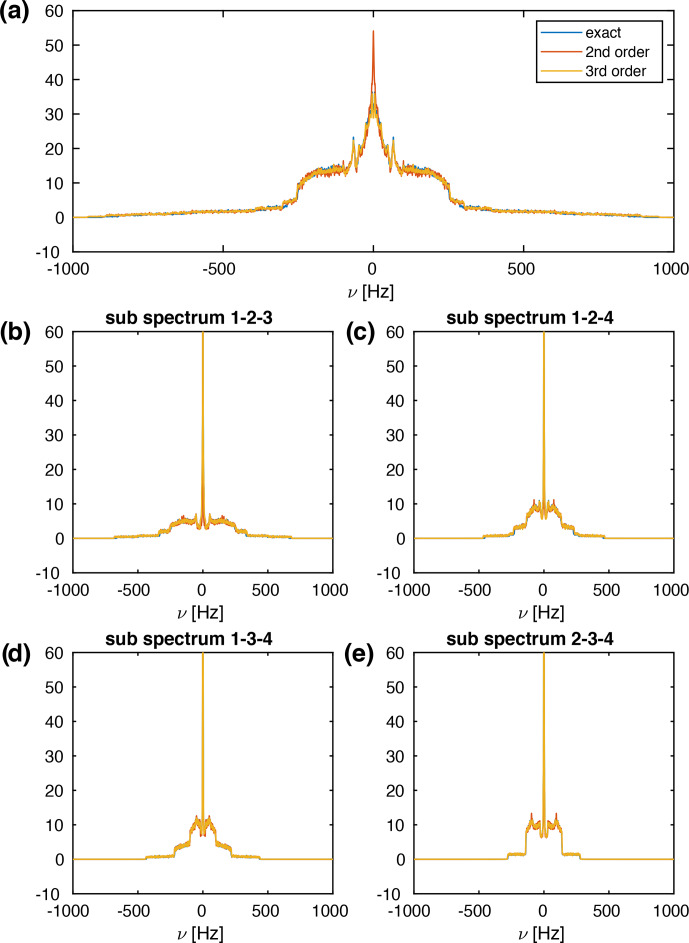
**(a)** Calculated MAS spectra for a four-spin system at a spinning frequency of 50 kHz using different approximations of the effective Hamiltonian. All spectra were processed with an exponential line broadening of 1 Hz. The spin-system parameters are $r_{1}=(0,0,0)$ Å, $r_{2}=(0,0,1.75)$ Å, $r_{3}=(3.0,0,0)$ Å, $r_{4}=(3.5,0.0,2.0)$ Å, $\delta_{12}/(2\pi )=-44\;826$ Hz, $\delta_{13}/(2\pi )=-8898$ Hz, $\delta_{14}/(2\pi )=-3667$ Hz, $\delta_{23}/(2\pi )=-5734$ Hz, $\delta_{24}/(2\pi )=-5561$ Hz, $\delta_{34}/(2\pi )=-27420$ Hz. The Euler angles can be calculated from the coordinates given above. The spectral window was set to 2000 Hz with 20 000 data points leading to a digital resolution of 0.1 Hz. Ten thousand powder points were sampled according to the ZCW scheme [Bibr bib1.bibx14]. Panels **(b)**–**(e)** show the four three-spin sub-spectra that comprise the complete four-spin spectrum shown in panel **(a)**.

## 5 Experimental data

We also tried to experimentally characterize the line-position changes associated with the time evolution under the second-order Hamiltonian as expected from Eq. (9). As shown in Fig. [Fig Ch1.F6]a, such effects are only on the order of several Hertz and, therefore, hard to extract from experimental proton-detected solid-state NMR spectra. To enhance proton resolution to a degree that makes the observation of such small effects possible, we recorded ${}^{1}H$ MAS spectra of the crystalline compound ortho-phospho-L-serine previously studied by solid-state NMR [Bibr bib1.bibx15] at MAS frequencies ranging from 70 to 158 kHz and extracted the apparent isotropic chemical shifts of the methylene group ${\mathrm{CH}}_{2}$ protons and the CαH proton by line-shape deconvolution of each spectrum using DMFIT4 [Bibr bib1.bibx30]. This becomes possible due to decreasing coherent line-broadening contributions to the proton line width at fast MAS [Bibr bib1.bibx45]. The chemical-shift deviations for the three protons from the values obtained at 158 kHz are given in Fig. [Fig Ch1.F8] and indeed reveal the theoretically predicted dependence on the MAS spinning frequency. We also simulated the line-shift changes by using a simplified three-spin system with parameters relying on the crystal structure (CSD entry SERPOP03). Indeed, the experimentally observed trends can be reproduced reasonably well by these simple three-spin simulations taking only second-order effects into account. However, an even more accurate experimental determination of such an effect is still to some extent limited by the residual broadening of proton resonances in this crystalline compound (see Fig. [Fig Ch1.F8]c). The resonances of ortho-phospho-L-serine reported in the experimental section (the methylene ${\mathrm{CH}}_{2}$ and the CαH protons) do not show a measurable temperature dependence of the chemical-shift values as described recently [Bibr bib1.bibx28].

**Figure 8 Ch1.F8:**
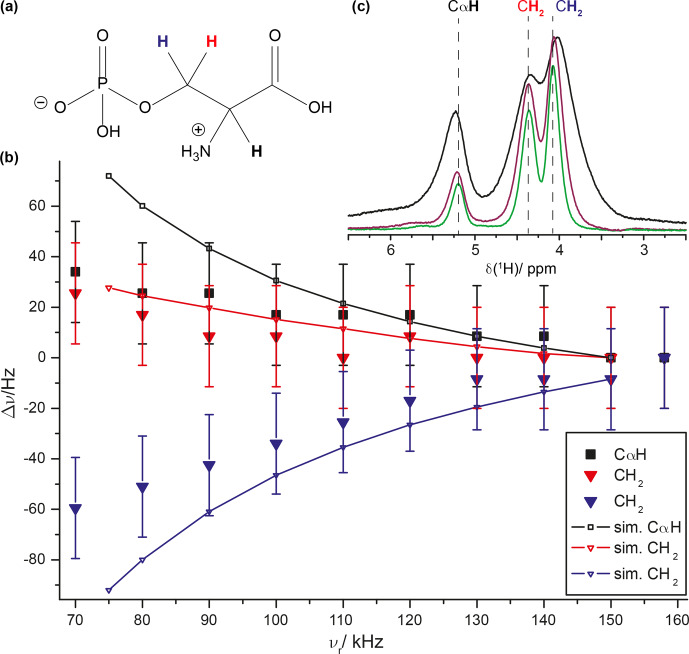
MAS dependence of ${}^{1}H$ line positions in ortho-phospho-L-serine (for the chemical structure, see panel **a**). **(b)** The filled symbols indicate experimental data with a constant error of 20 Hz. The two ${\mathrm{CH}}_{2}$ protons are shown in red (high ppm peak) and blue (low ppm peak); the CαH proton is shown in black. The simulations were based on a three-spin system with $r_{12}$ = 1.60 Å, $r_{23}=r_{13}$ = 2.35 Å, and θ(1,2,3)=θ(2,1,3)=70 ${}^{\circ }$ . Three ${}^{1}H$ spectra recorded with MAS frequencies of 70 kHz (black), 120 kHz (purple), and 158 kHz (green) are shown in panel **(c)**. The dashed lines indicate the isotropic ${}^{1}H$ chemical shift at 158 kHz MAS. The experiments were performed at a static magnetic field of 20 T.

## 6 Conclusions

We have shown through numerical simulations using various orders of effective Hamiltonians that second-order dipolar contributions under MAS lead to an MAS dependence of the line position and dominate the residual line broadening in dipolar-coupled homonuclear spin systems. Third-order terms do not play a significant role for the residual line width but change the line shape close to the center of the line. Fourth-order terms were not explicitly calculated but were shown to be contributing to the line width at MAS frequencies below 50 kHz in strongly coupled proton spin systems. Without chemical-shift differences we observe a clear $\omega_{r}^{-1}$ dependence of the residual line width under MAS in three-spin as well as larger spin systems above 50 kHz MAS. The implementation of chemical-shift differences leads to a change in this spinning-frequency dependence in three-spin systems. The clear $\omega_{r}^{-1}$ dependence changes to a spinning-frequency dependence somewhere between $\omega_{r}^{-1}$ and $\omega_{r}^{-2}$ depending on the details of the involved chemical shifts. Looking at larger spin systems with chemical-shift differences is more complex using the approach used here since the separation of the lines becomes more difficult due to the larger number of combination lines. We are currently working on this problem that is beyond the scope of this paper.

## Supplement

10.5194/mr-2-499-2021-supplement The supplement related to this article is available online at: https://doi.org/10.5194/mr-2-499-2021-supplement.

## Data Availability

The simulation data and the processing and plot scripts for all figures are available at https://doi.org/10.3929/ethz-b-000490555 (Chavez et al., 2021).
