# Supplementary material for: Residual dipolar line width in magic-angle spinning proton solid-state NMR
Source: Magn Reson (Gott). 2021 Jul 1;2(1):499–509. doi: 10.5194/mr-2-499-2021 (PMC10539731; doi:10.5194/mr-2-499-2021)
Supplement: The supplement related to this article is available online at: https://doi.org/10.5194/mr-2-499-2021-supplement. [file mr-2-499-supplement.pdf]

Supplement of Magn. Reson., 2, 499–509, 2021
https://doi.org/10.5194/mr-2-499-2021-supplement
© Author(s) 2021. CC BY 4.0 License.

Supplement of

Residual dipolar line width in magic-angle spinning proton solid-state NMR

Matías Chávez et al.

Correspondence to: Matthias Ernst (maer@ethz.ch) and Beat H. Meier (beme@ethz.ch)

The copyright of individual parts of the supplement might differ from the article licence.

S1 Effective coupling frequencies of the second-order Hamiltonian

$$\hat{\bar{\mathcal{H}}}^{(2)} = \frac{1}{2}\sum_{n\neq 0} \frac{\left[\hat{\mathcal{H}}^{(n)}, \hat{\mathcal{H}}^{(-n)}\right]}{n\omega_{\mathrm{r}}} = \sum_{p,q,r} \omega_{pqr}^{(\mathrm{eff})} \hat{I}_{pz}\left(\hat{I}_q^+ \hat{I}_r^- - \hat{I}_q^- \hat{I}_r^+\right) \tag{S1}$$

with

$$\omega_{pqr}^{(\mathrm{eff})} = \sum_{n\neq 0} \frac{1}{2\,n\,\omega_{\mathrm{r}}}\left(\frac{1}{2}\omega_{pq}^{(n)}\omega_{pr}^{(-n)} + \omega_{pq}^{(n)}\omega_{qr}^{(-n)} + \omega_{pr}^{(n)}\omega_{qr}^{(-n)}\right) \tag{S2}$$

S2 Effective coupling frequencies of the third-order Hamiltonian

$$\begin{aligned}\hat{\bar{\mathcal{H}}}^{(3)} =& \frac{1}{2}\sum_{n\neq 0} \frac{\left[\left[\hat{\mathcal{H}}^{(n)}, \hat{\mathcal{H}}^{(0)}\right], \hat{\mathcal{H}}^{(-n)}\right]}{(n\omega_{\mathrm{r}})^2} + \frac{1}{3}\sum_{k,n\neq 0} \frac{\left[\hat{\mathcal{H}}^{(n)}, \left[\hat{\mathcal{H}}^{(k)}, \hat{\mathcal{H}}^{(-k-n)}\right]\right]}{n\omega_{\mathrm{r}} k\omega_{\mathrm{r}}} \\ =& \sum_p \omega_p^{(\mathrm{eff})}\hat{I}_{pz} + \sum_{p,q}\omega_{pq,z}^{(\mathrm{eff})}\left(2\hat{I}_{pz}\hat{I}_{qz}\right) - \omega_{pq}^{(\mathrm{eff})}\left(\hat{I}_{px}\hat{I}_{qx} + \hat{I}_{py}\hat{I}_{qy}\right) + \sum_{p,q,r}\omega_{pqr}^{(\mathrm{eff})}\hat{I}_{pz}\left(\hat{I}_q^+\hat{I}_r^- + \hat{I}_q^-\hat{I}_r^+\right) \\ &+ \sum_{p,q,r,s}\omega_{pqrs,z}^{(\mathrm{eff})}\hat{I}_{pz}\hat{I}_{qz}\left(\hat{I}_r^+\hat{I}_s^- + \hat{I}_r^-\hat{I}_s^+\right) + \sum_{p,q,r,s}\omega_{pqrs,xy}^{(\mathrm{eff})}\left(\hat{I}_p^+\hat{I}_q^-\hat{I}_r^+\hat{I}_s^- + \hat{I}_p^-\hat{I}_q^+\hat{I}_r^-\hat{I}_s^+\right)\end{aligned} \tag{S3}$$

$$\omega_p^{(\mathrm{eff})} = \frac{5}{6}\sum_{p\neq q}\sum_{n\neq 0}\frac{\omega_{pq}^{(n)}\omega_{pq}^{(-n)}(\omega_q^{(0)} - \omega_p^{(0)})}{(n\omega_r)^2} \tag{S4}$$

$$\omega_{pq}^{(\mathrm{eff})} = \sum_{\substack{n,k\neq 0\\ n\neq k}}\sum_r \frac{5}{8}\left(\mathrm{sign}(nk)\omega_{pq}^{(-n-k)}\omega_{pr}^{(n)}\omega_{pr}^{(k)}\right) - \frac{1}{2}\left(\frac{\mathrm{sign}(nk)}{\max(|n|,|k|)}\omega_{pq}^{(-n-k)}\omega_{pr}^{(n)}\omega_{qr}^{(k)}\right) \tag{S5}$$

$$\omega_{pq,z}^{(\mathrm{eff})} = \sum_{\substack{n,k\neq 0\\ n\neq k}}\sum_r \frac{\mathrm{sign}(nk)}{4}\left(\omega_{pq}^{(-n-k)}\omega_{pr}^{(n)}\omega_{pr}^{(k)} + \omega_{pr}^{(n)}\omega_{qr}^{(k)}\omega_{qr}^{(-n-k)}\right) - \frac{1}{8}\left(\omega_{pq}^{(n)}\omega_{pr}^{(k)}\omega_{qr}^{(-n-k)}\right) \tag{S6}$$

$$\omega_{pqr}^{(\mathrm{eff})} = \frac{6}{5}\sum_{n\neq 0}\frac{\omega_{pq}^{(n)}\omega_{pr}^{(-n)}\omega_q^{(0)} - 2\,\omega_{qp}^{(n)}\omega_{pr}^{(-n)}\omega_r^{(0)}}{(n\omega_r)^2} \tag{S7}$$

All terms listed in Eqs. (S4)-(S7) are the terms that appear in a three-spin system. In a four-spin system, additional four-spin terms (the last two terms in Eq. (S3)) appear. The analytical expressions for these terms have been calculated in Mathematica but are too complex to print. For the numerical simulations, the commutator and double commutator expressions of Eqs. (S1) and (S3) were implemented using the Fourier coefficients $\hat{\mathcal{H}}^{(n)}$ of the Hamiltonians.

S3 Additional Simulations for a Three-Spin System

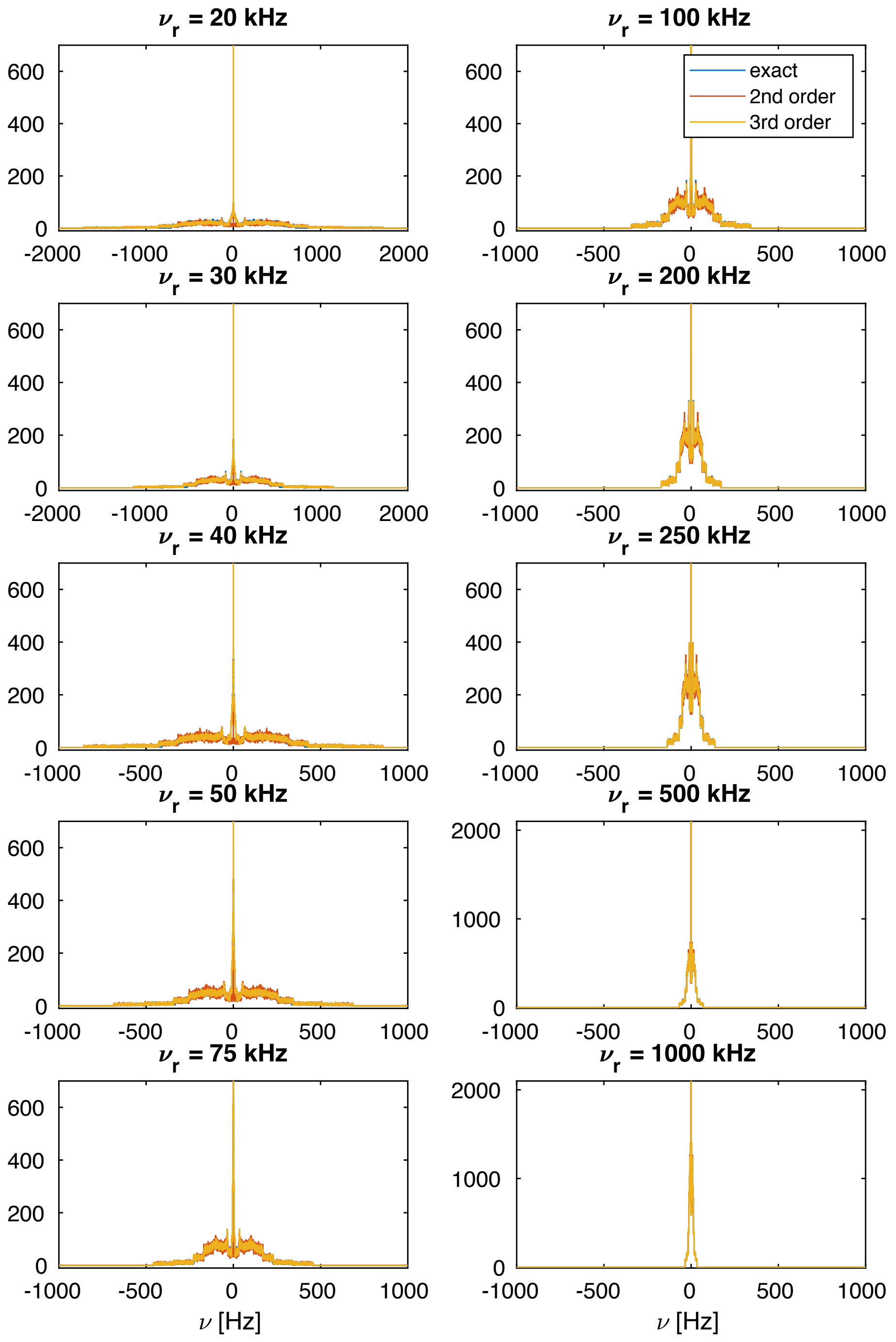

Figure S1: Calculated MAS spectra at a spinning frequencies between 20 kHz and 1 MHz using different approximations of the effective Hamiltonian. All spectra were processed with an exponential line broadening of 1 Hz. The spin-system parameters are: $\delta_{12}/(2\pi) = -45000$ Hz, $\delta_{13}/(2\pi) = -8898$ Hz, $\delta_{23}/(2\pi) = -5750$ Hz, $\theta_{13} = 90°$, $\theta_{23} = 120°$. The spectral window was set between 200 and 4000 Hz with 20000 data points leading to a digital resolution of ranging from 0.01 to 0.2 Hz. One hundred thousand powder points were sampled according to the ZCW scheme for each of the spectra (Cheng et al., 1973).

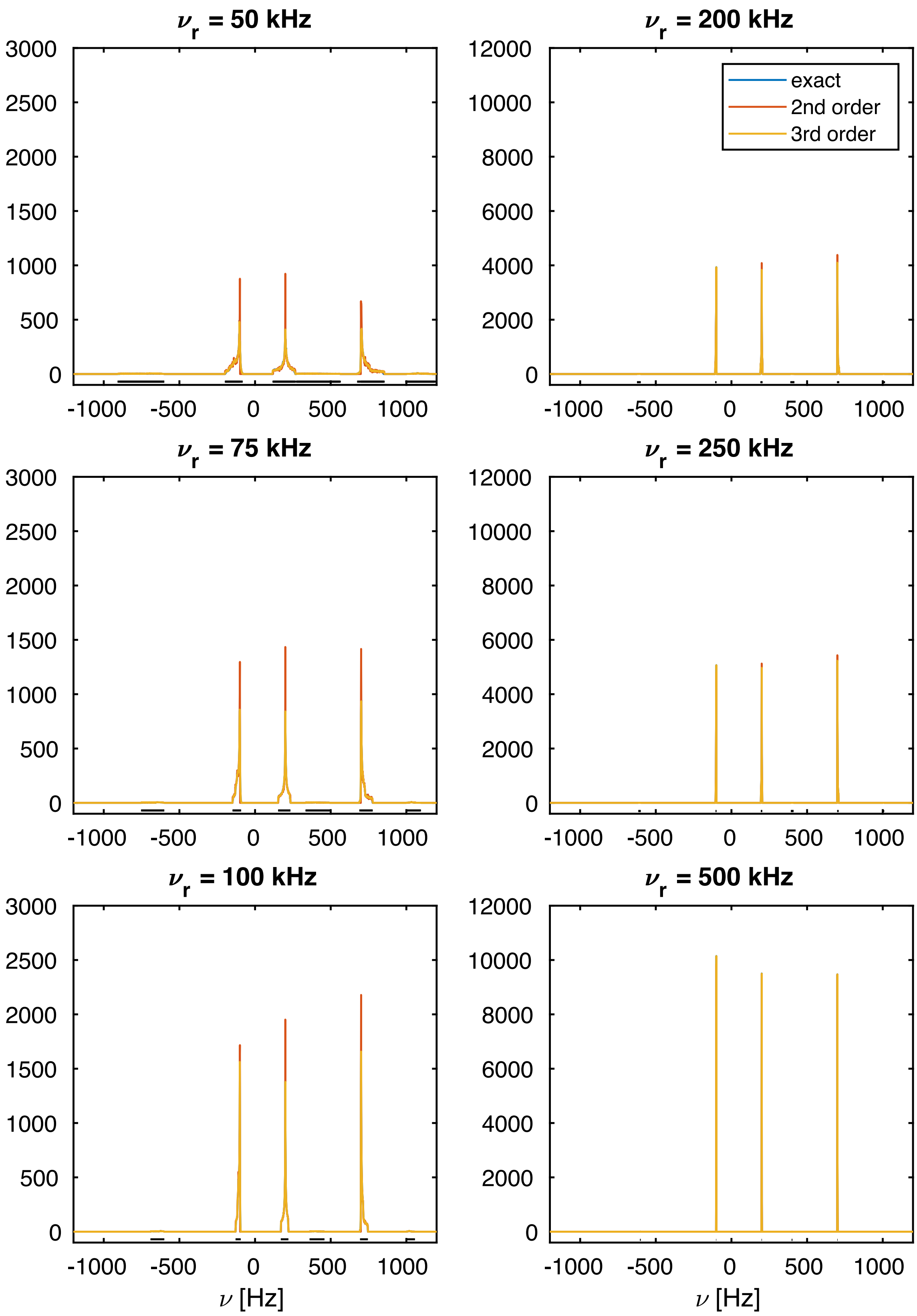

Figure S2: Calculated MAS spectra at a spinning frequencies between 50 and 500 kHz using different approximations of the effective Hamiltonian. The spin-system parameters are: $\delta_{12}/(2\pi) = -45000$ Hz, $\delta_{13}/(2\pi) = -8898$ Hz, $\delta_{23}/(2\pi) = -5750$ Hz, $\theta_{13} = 90°$, $\theta_{23} = 120°$. The spectral window was set between 200 and 4000 Hz with 20000 data points leading to a digital resolution ranging from 0.01 to 0.2 Hz. One hundred thousand powder points were sampled according to the ZCW scheme for each of the spectra (Cheng et al., 1973).

S4 Additional Simulations for a Four-Spin System

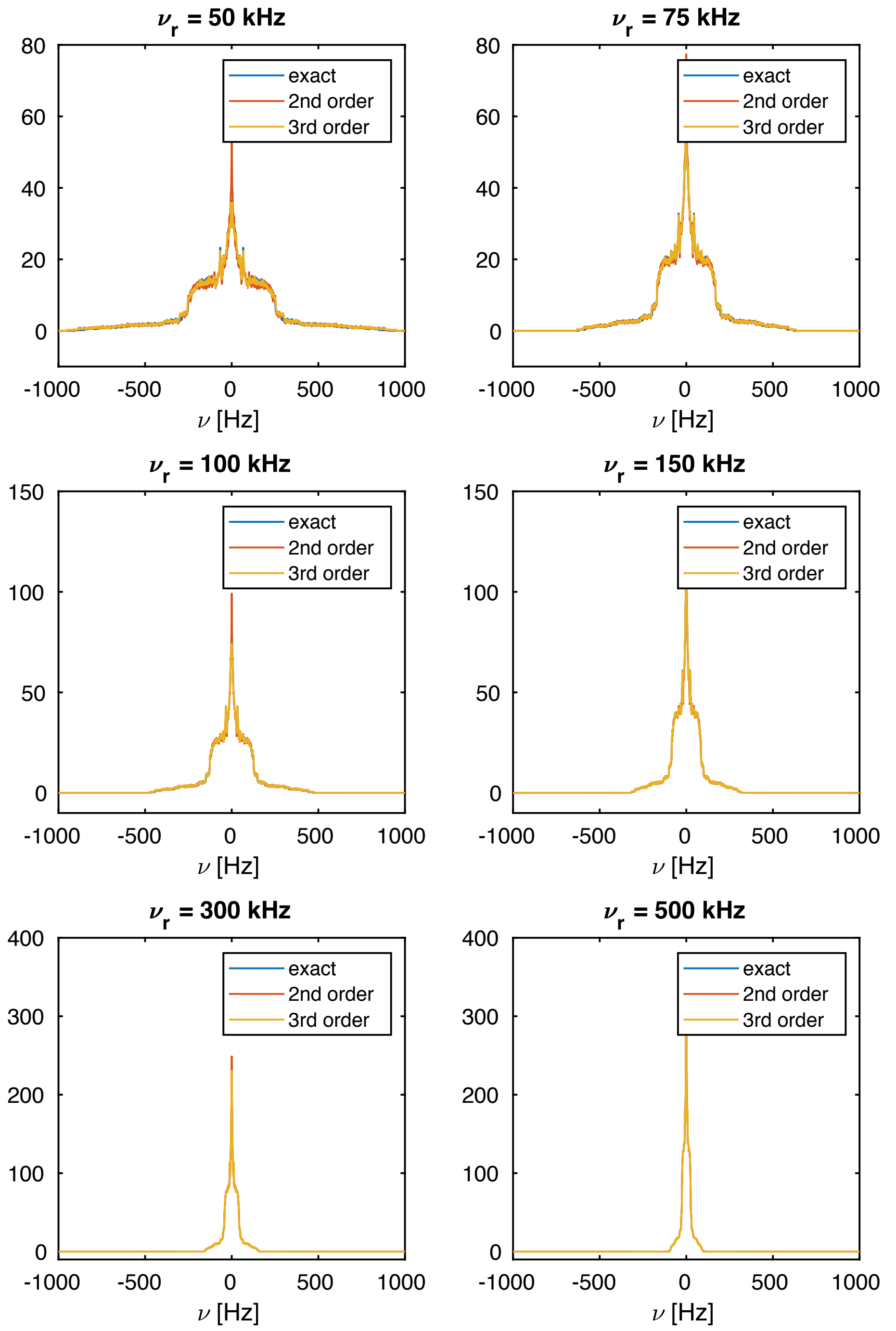

Figure S3: Calculated MAS spectra for a four-spin system at spinning frequencies between 50 and 500 kHz using different approximations of the effective Hamiltonian. All spectra were processed with an exponential line broadening of 1 Hz. The spin-system parameters are: $\vec{r}_1 = (0, 0, 0)$Å, $\vec{r}_2 = (0, 0, 1.75)$Å, $\vec{r}_3 = (3.0, 0, 0)$Å, $\vec{r}_4 = (3.5, 0.0, 2.0)$Å$\delta_{12}/(2\pi) = -44826$ Hz, $\delta_{13}/(2\pi) = -8898$ Hz, $\delta_{14}/(2\pi) = -3667$ Hz, $\delta_{23}/(2\pi) = -5734$ Hz, $\delta_{24}/(2\pi) = -5561$ Hz, $\delta_{34}/(2\pi) = -27420$ Hz. The Euler angles can be calculated from the coordinates given above. The spectral window was set to 2000 Hz with 20000 datapoints leading to a digital resolution of 0.1 Hz. Ten thousand powder points were sampled according to the ZCW scheme (Cheng et al., 1973).

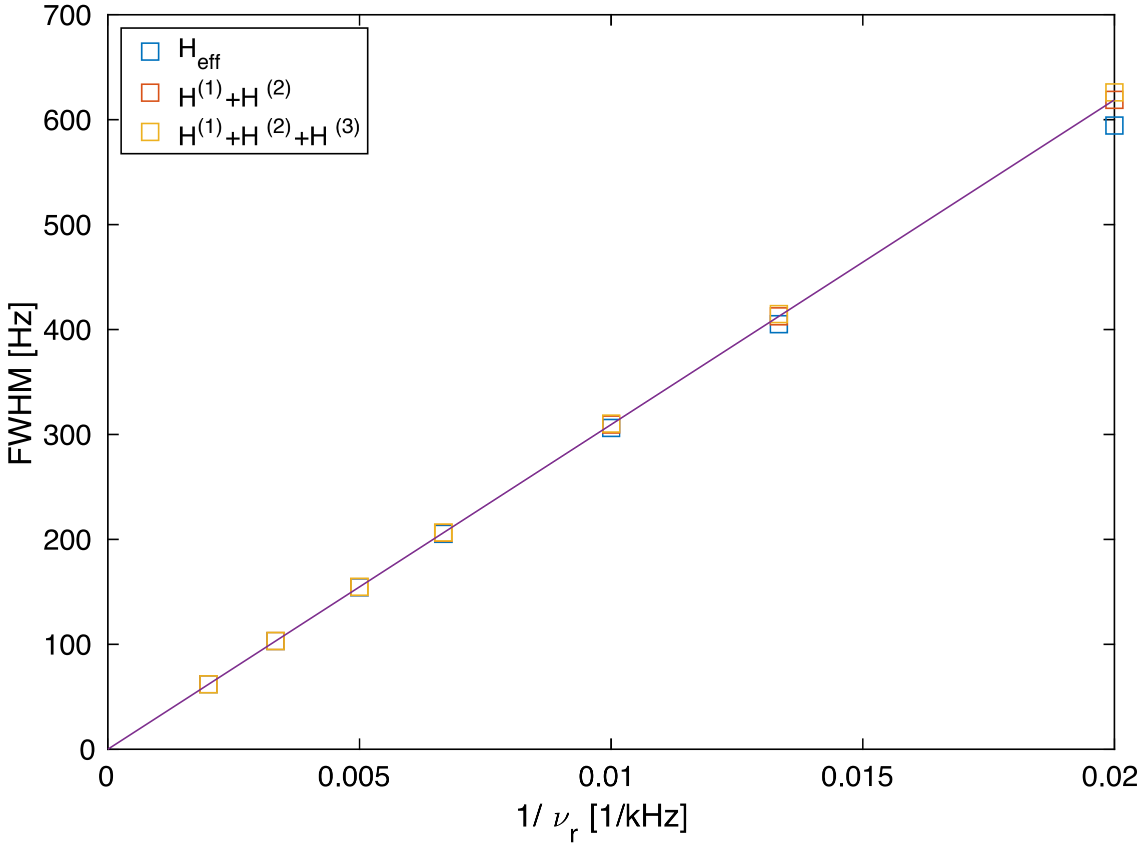

Figure S4: Line width (full-width half max) of a Gaussian line with the same second moment as the four-spin powder line shapes shown in the SI Fig. 3 (FWMH $= 2\sqrt{2\log(2)M_2}$). The line width obtained from second-order and third-order effective Hamiltonian correlates almost perfectly linear with ω_r^{-1} while the line width obtained from the exact effective Hamiltonian shows some deviations at 50 kHz. This deviation (narrower line width) is the contribution of the fourth-order term as discussed in the main paper.

S5 Additional Simulations for a Five-Spin System

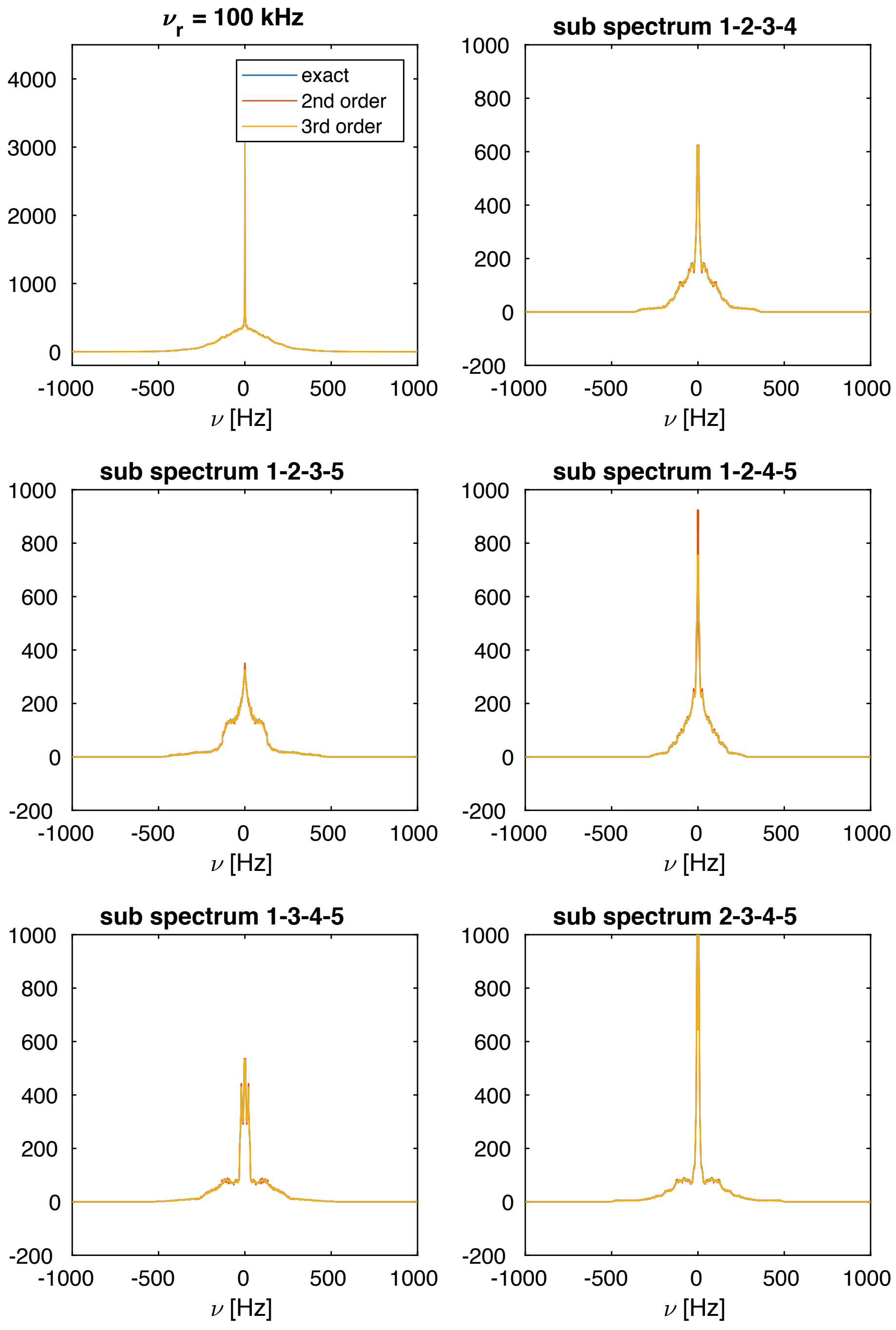

Figure S5: Calculated MAS spectra for a five-spin system at a spinning frequency of 100 kHz using different approximations of the effective Hamiltonian. All spectra were processed with an exponential line broadening of 1 Hz. The spin-system parameters are: $\vec{r}_1 = (0, 0, 0)$Å, $\vec{r}_2 = (0, 0, 1.75)$Å, $\vec{r}_3 = (3.0, 0, 0)$Å, $\vec{r}_4 = (3.5, 2.0, 2.0)$Å, $\vec{r}_5 = (3.5, -0.25, 2.0)$Å, $\delta_{12}/(2\pi) = -44828$ Hz, $\delta_{13}/(2\pi) = -8898$ Hz, $\delta_{14}/(2\pi) = -2636$ Hz, $\delta_{15}/(2\pi) = -3646$ Hz, $\delta_{23}/(2\pi) = -5734$ Hz, $\delta_{24}/(2\pi) = -3646$ Hz, $\delta_{25}/(2\pi) = -5519$ Hz, $\delta_{34}/(2\pi) = -10138$ Hz, $\delta_{35}/(2\pi) = -26826$ Hz, $\delta_{45}/(2\pi) = -21091$ Hz. The angles can be calculated from the coordinates given above.The spectral window was set to 2000 Hz with 20000 datapoints leading to a digital resolution of 0.1 Hz. Ten thousand powder points were sampled according to the ZCW scheme (Cheng et al., 1973). (b)-(f) Calculated MAS spectra of the five four-spin sub spectra comprising the complete five-spin spectrum.

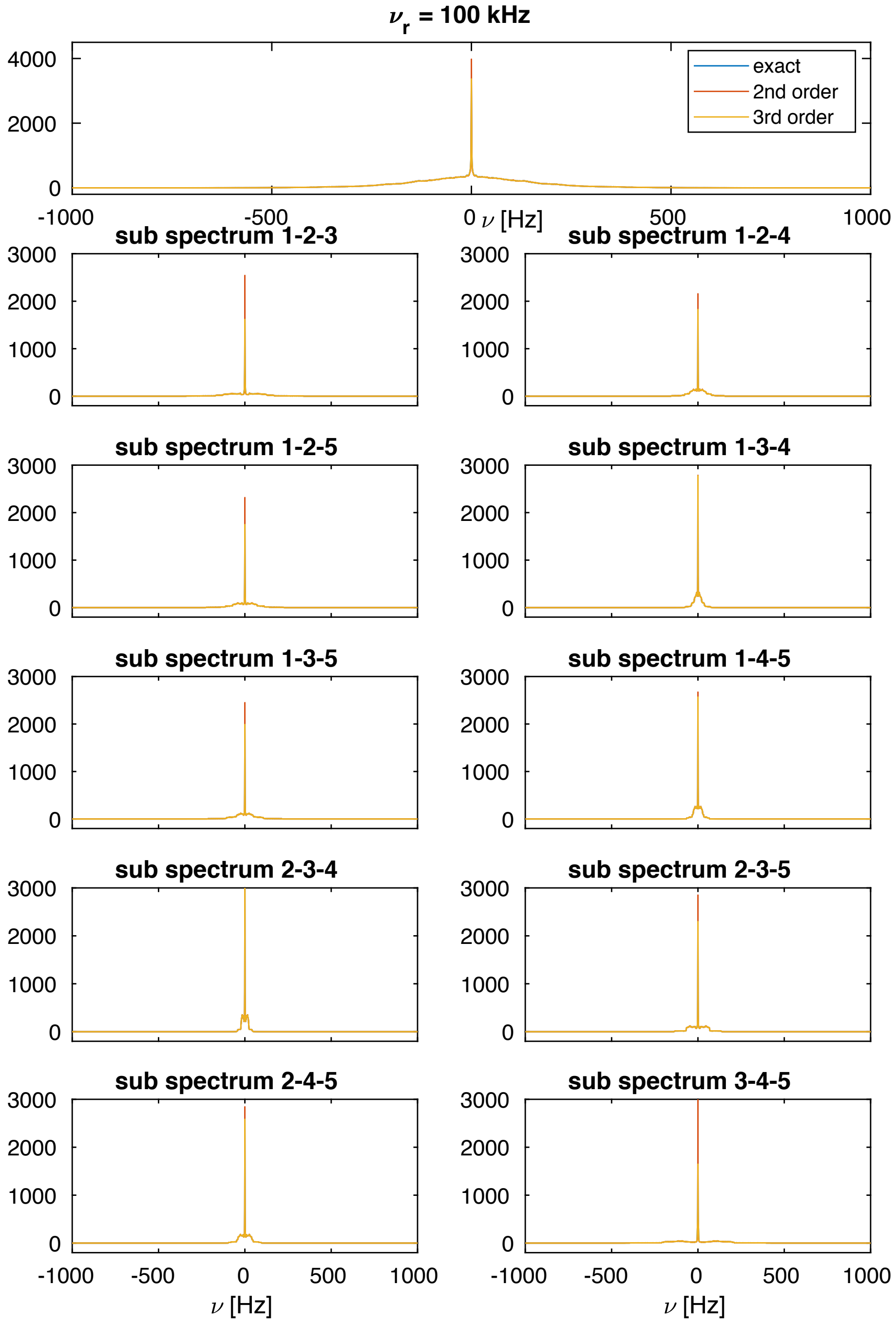

Figure S6: Calculated MAS spectra for a five-spin system at a spinning frequency of 100 kHz using different approximations of the effective Hamiltonian. All spectra were processed with an exponential line broadening of 1 Hz. The spin-system parameters are: $\vec{r}_1 = (0,0,0)$Å, $\vec{r}_2 = (0,0,1.75)$Å, $\vec{r}_3 = (3.0,0,0)$Å, $\vec{r}_4 = (3.5,2.0,2.0)$Å, $\vec{r}_5 = (3.5,-0.25,2.0)$Å, $\delta_{12}/(2\pi) = -44828$ Hz, $\delta_{13}/(2\pi) = -8898$ Hz, $\delta_{14}/(2\pi) = -2636$ Hz, $\delta_{15}/(2\pi) = -3646$ Hz, $\delta_{23}/(2\pi) = -5734$ Hz, $\delta_{24}/(2\pi) = -3646$ Hz, $\delta_{25}/(2\pi) = -5519$ Hz, $\delta_{34}/(2\pi) = -10138$ Hz, $\delta_{35}/(2\pi) = -26826$ Hz, $\delta_{45}/(2\pi) = -21091$ Hz. The angles can be calculated from the coordinates given above.The spectral window was set to 2000 Hz with 20000 datapoints leading to a digital resolution of 0.1 Hz. Ten thousand powder points were sampled according to the ZCW scheme (Cheng et al., 1973). (b)-(k) Calculated MAS spectra of the eleven three-spin sub spectra comprising the complete five-spin spectrum.

References

Cheng, V. B., Suzukawa, H. H., and Wolfsberg, M.: Investigations of a Nonrandom Numerical-Method for Multidimensional Integration, J. Chem. Phys., 59, 3992–3999, 1973.
